# Supplementary material for: Identification of Key Genes and Biological Pathways Associated with Skeletal Muscle Maturation and Hypertrophy in Bos taurus, Ovis aries, and Sus scrofa
Source: Animals (Basel). 2022 Dec 8;12(24):3471. doi: 10.3390/ani12243471 (PMC9774933; doi:10.3390/ani12243471)
Supplement: Supplementary file 1 [file animals-12-03471-s001.zip › animals-2074792-supplementary.pdf]

animals

MDPI

Supplementary Materials: The following supporting information can be downloaded at: www.mdpi.com/xxx/s1,

Table S1. Name of common genes identified between 3 species using Venn diagram.

Cattle, sheep, pig	CXCR4,DTNBP1,GCLM,THRB,PPARGC1A,FST,LXN,TYMS,RPL9,HBB,ARG2,JUP,UCP3,COL1A2,PHKB,ISG15,DGAT2,APOA1,PYGM,PLIN2,MYOD1,NAMPT,POSTN,EPAS1,ME1,CKAP4,MID1IP1,JAG1,SDC4,ILF3,AIMP2,EEF1A1,IGF2,MB,DDIT3,BCAT1

Table S2. Name of common genes identified between cattle and sheep using Venn diagram.

Cattle, sheep	NOS2,COL1A1,TNXB,PRND,PROS1,SLC11A1,THBS1,IGFBP3,MACF1,IRF1,FBLIM1,TNFRSF11B,AQP4,SNX10,SH3BGRL,ARPP19,SLC4A7,BUB1,COL3A1,CD3G,CD96,RCL1,HYOU1,GPRC5C,SELP,FCGR3A,SLC2A1,PTGER4,CD14,CD74,TP53I11,MAPRE2,MADCAM1,LYZ,MYOG,NDRG4,MANSC1,INHA,CTSA,CD3E,ARL1,HIF3A,S100A14,SLC7A8,JMJD6,HOMER1,SASH3,IFI30,CATHL3,CD52,ICAM1,QSOX1,LAMA4,TGFBI,PSMB9

Table S3. Name of common genes identified between cattle and pis using Venn diagram.

Cattle, pig	ABRA,GNL3,TMSB10,CCNI,CCNB1,LYAR,UFSP1,CCDC34,CDC25B,FGGY,TMEM52,C1QTNF3,NEIL1,APOE,ID1,ZNHIT2,HSF2,CD53,NOP58,ASAH1,RGS14,ARMC6,HP,PIGL,SCAMP3,KIAA0930,RCAN1,LIMD2,POLR1A,CA9,IFNAR1,B2M,HCFC1R1,IL18,MCM5,MELK,MAN2C1,TMEM132A,RPL27,ALAS1,PSMA5,ABI3,ACOX3,CKS2,ASPN,CSDC2,PDLIM1,ADHFE1,SERPINI1,CCNB2,LGALS3,FCF1,TAGLN3,OBFC1,ENPEP,RPL3,TCAP,MTHFD1L,BHLHE40,HDAC11,TNIP1,ODC1,HIP1R,LRRC1,CDKN1A,NPM3,RARRES1,HRC,CENPI,CRYL1,LMCD1,DUS1L,EIF3E,KDELR2,CKMT2,PLP1,TNPO2,FBXL4,FKBP11,SLC4A4,DDX56,ARL5A,CCT6A,RNASE4,MAPK8IP1,ANKZF1,PSPH,TBC1D19,PTN,HIVEP1,HSPB6,NUBP2,ID3,CAPN3,DEPDC7,TSPAN3,THOC5,EPDR1,HMOX2,TAGLN2,CACNB3,PLTP,AKIRIN2,ACBD5,TTLL1,ANKRD28,SYN1,LIPE,ZNF382,DBF4,AHCY,FAM69B,CDKN2D,SMPD1,MAPRE3,VLDLR,RNF19B,VIM,KIF21A,AMPD1,HEXB,RRS1,PYCR1,PL

	CD4,CDC20,RCAN3,GCHFR,UBD,S100A1,DRG1,AIF1,IFRD1,SKA1,CAMK2G,PPAN,RPL26,RGS5,FOSL2,PRDX3,WDR46,OCIAD2,ERP29,TGFB3,B3GALNT1,GPR183,HIVEP2,MCOLN1,NLN,UBE2C,NQO1,RPL5,TRAPPC1,CYB5R1,MUL1,AGPAT2,WIPI1,CYP27A1,CCDC80,HTATIP2,NEMF,PAMR1,RRM2,IGF1R,EFEMP2,CLEC1A,PLBD1,SPARCL1,ANKRD29,HIF1A,S100A10,HSPH1,DHDH,ME3,GPD2,KCNB1,COL6A3,PABPC1,RNF130,DCTD,SEPHS2,SRSF5,ACOX2,POLR3D,SAR1B,CENPF,RAB2A,BYSL,PEBP4,NDUFS1,RNF14,FXYD6,SDAD1,TBL3,CLTA,KPNA2,MACROD1,BOP1,POLR1E,RAB3A,PDZRN3,NPM1,TMEM117,EFHC1,EIF3M,ACTC1,PTGES,MFAP5,WDR12,FN1,LOXL1,IFI16,TMEM25,CEBPD,RAD1,CYB561,THY1,MAP1A,CD200,CCL4,TPPP3,RBM3,PTTG1,SSR4,FN3K,COMMD8,CCDC167,ASB11,CNN2,LUM,GLO1,DYRK3,TSPAN12,PPP3CB,AMFR,ISLR,BLVRA,PIP4K2A,FSCN1,IGFBP6,CFL1,MYF6,F2R,FBP1,KBTBD4,ALG8,DLGAP5,AKAP8L,SEC11A,CCL5,SH3BGRL3,MX1,LOXL4,TGFBR3,CPNE1,TMEM120A,BNIP3L,CDKN2C,AGPAT5,ACP6,SEPW1,IGF2R,SCMH1,GYG1,MTCH2,LACTB,RPS9,HNRNPA1,CFD,ART5,PGK1,PRCP,DIO3,TMOD4,SYNE2,FBP2,RFC3,SLC44A2,UCHL1,TSSC1,LGALS1,SLC2A3,GPI,ASF1B,CAMK2D,TNNI3,SPTAN1,CITED1,STBD1,MAP2K6,CBLB,C5H12orf57,WARS,ASPA,PCYOX1,GABARAPL1,IL15,PDCD2L,TFRC,BCOR,NSUN3,EXOSC5,S100A11,ANXA5,EPHX2,GAR1,RXRG,TPM4,OPTN,CDCA3,S100A4,CHRNA1,SLC2A12,HOMER2,POLE2,IFIT1,SAP30,JARID2,THBS2,CA2,SORD,PADI2,DST,NPPC,RUSC2,CMBL,MPDZ,SDHC,CCL21,MSTN,RNASEH2A,RNF40,RSAD2,ALS2CL,CDK5,IRF8,LRRC28,ATP2A2,ADRBK2

Table S4. Name of common genes identified between pig and sheep using Venn diagram.

Pig, sheep	BTG2,ARL6IP1,PSMD11,STAT5A,NDRG2,MYF5,IGFBP2,CD93,FOXO1,GUCA2A,GNLY,ABHD5,LMNB1,OCLN,MMP14,MXRA7,ABCF1,EML4,CDC42EP5,CD247,GNG11,PEG3,PDIA6,LPL,NREP,ALB,RCBTB2,GSTA1,MDFIC,CRYAB,FZD2,CD40,TAP1,ETS2,SCD5,PPTC7,LEF1,MAN1C1,MPZL1,TIE1,RIMKLB,UBE2B,CPQ,MCL1,HMGB2,CASP3,RGS2,ABCG2,CLSTN1,PREB,SDC1,ACTB,DAP,CIRBP,GPX3,COL5A1,FBXO32,CRKL,SSTR5,APP,ARNT2,IGF1,MITF,PON3,ENDOG,ARL2BP,PFKL,ZFP36,COL5A2,KIT,BAG3,CTSK,TINAGL1,STAT1,CREM,ANGPTL4,HMGN2,SYNCRIP,SLC25A20,PSMD4,IFT27,

	ZFAND5,MYADM,MYL4,SQSTM1,CALML4,TSC22D3,HSPA4,CKS1B,SLPI,VDAC1,ATF4,ANGPT1,EIF4G1,HSP90AA1,ANGPT2,CD34,KDR,DOCK9,DNMT1,CLCN5,COL2A1,GLUL,RASSF2,SLC25A33,NFKBIB,SERPINE1,CCND2,NNT,ITGB3,ANKRD1,FKBP9,CEBPB

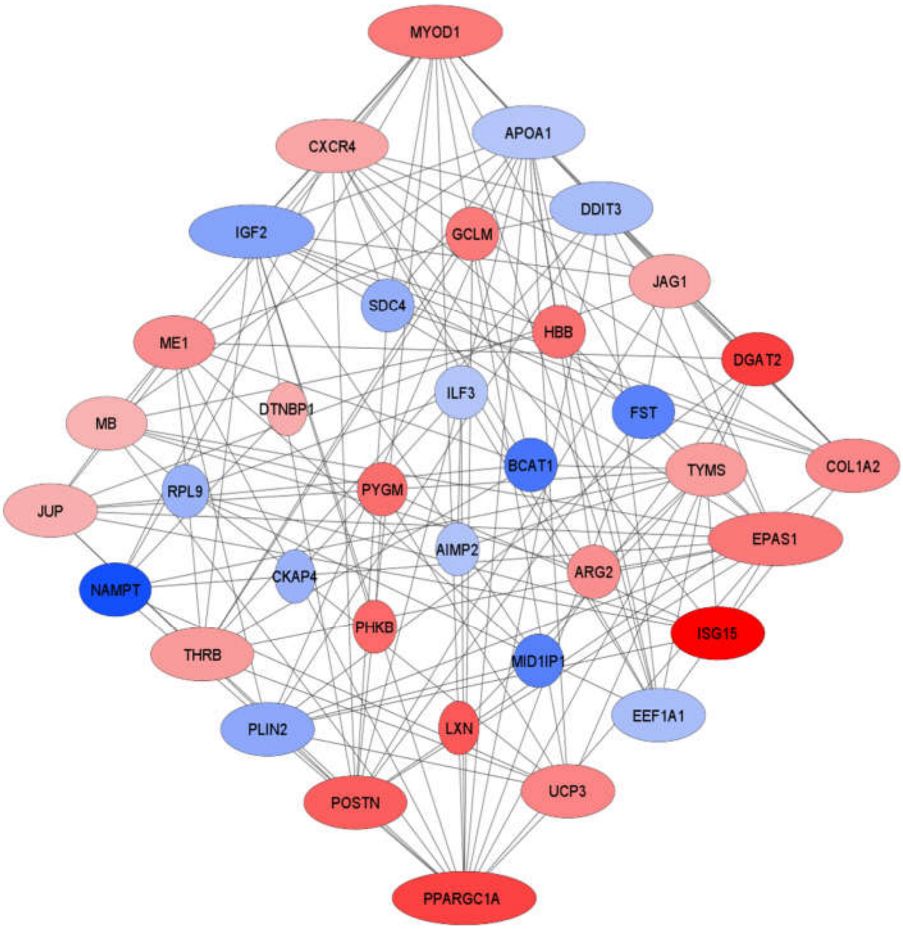

Figure S1. Differentially expressed genes with blue and red colors has been shown, blue showing low expression and red showing greater expression. The node size is proportional to the degree bigger than 12. The bigger size of the node means higher engagement of protein in the developmental process of skeletal muscle in cattle.

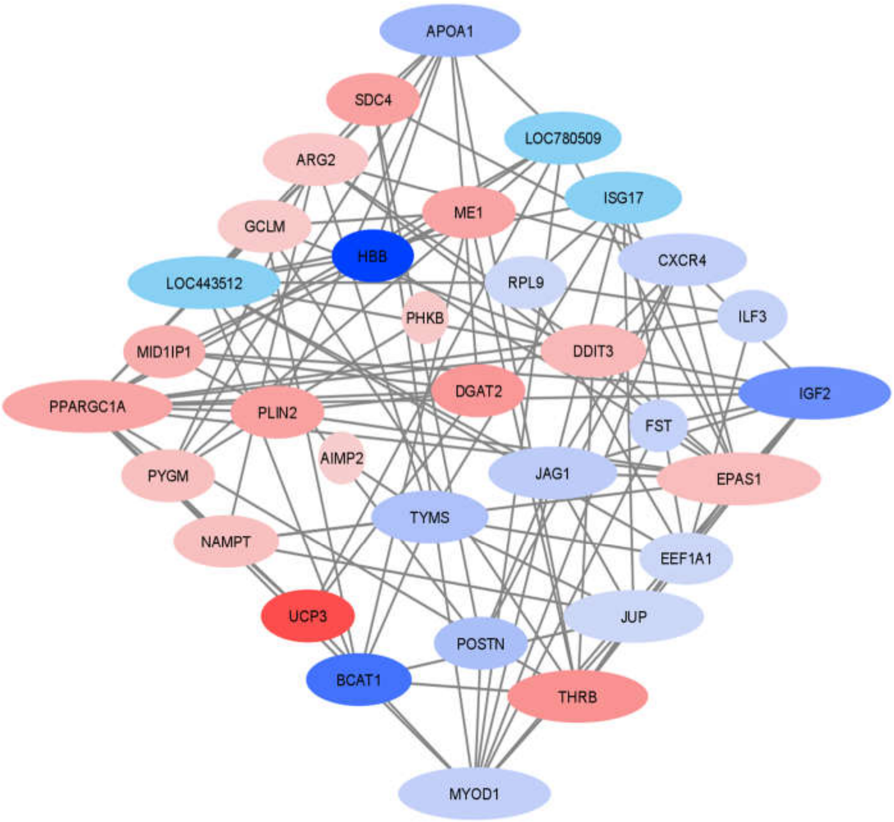

Figure S2. Differentially expressed genes with blue and red colors has been shown, blue showing low expression and red showing greater expression. The bigger size of the node means higher engagement of protein in the developmental process of skeletal muscle in sheep.

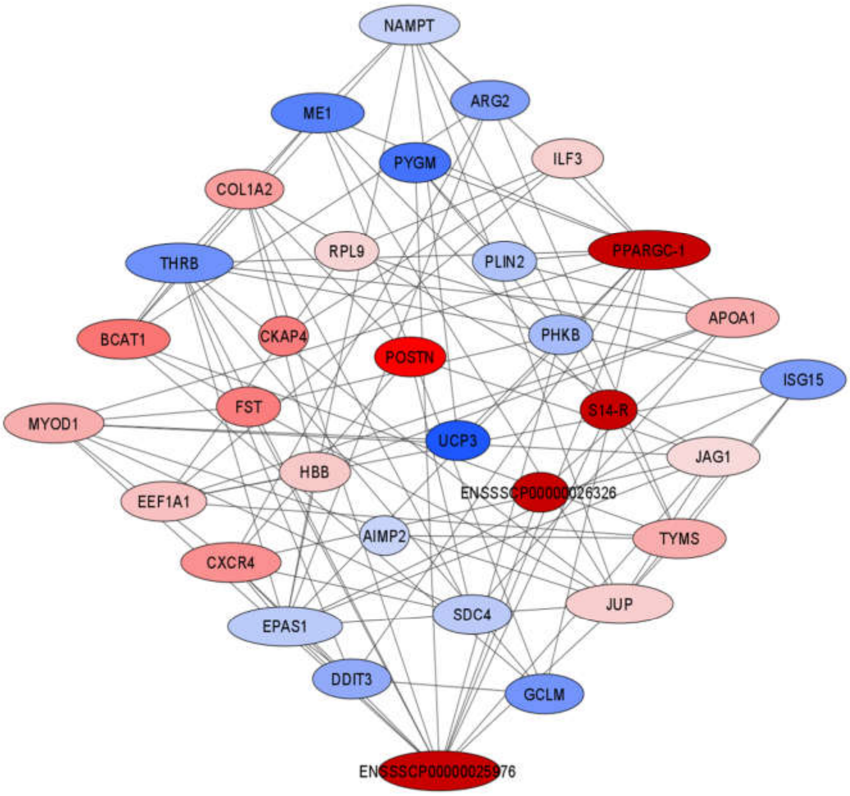

Figure S3. Differentially expressed genes with blue and red colors has been shown, blue showing low expression and red showing greater expression. The bigger size of the node means higher engagement of protein in the developmental process of skeletal muscle in pigs.

Table S5. Genes ranked by degree method in cattle.

Gene	Degree	Betweenness	Closeness
PPARGC1A	15.0	174.57031	0.625
MYOD1	14.0	76.05031	0.59322035
EPAS1	14.0	73.11194	0.59322035
IGF2	13.0	99.939415	0.546875
CXCR4	12.0	49.891876	0.5555556
APOA1	12.0	65.64227	0.5645161
POSTN	10.0	15.989016	0.5072464
DDIT3	10.0	35.881718	0.530303

THRB	10.0	38.24023	0.53846157
COL1A2	9.0	16.745413	0.5072464
PLIN2	9.0	31.412851	0.530303
UCP3	9.0	35.587242	0.53846157
JUP	9.0	57.627388	0.530303
EEF1A1	9.0	85.11197	0.53846157
ISG15	9.0	87.383766	0.546875
JAG1	8.0	5.740855	0.4861111
MB	8.0	21.683039	0.5072464
ME1	8.0	26.634432	0.46666667
TYMS	8.0	133.24516	0.5072464
NAMPT	7.0	87.734795	0.52238804
DGAT2	7.0	11.039767	0.4861111
FST	6.0	3.574481	0.47297296
SDC4	5.0	1.352381	0.43209878
HBB	5.0	3.8760684	0.4861111
ARG2	5.0	14.070007	0.46666667
GCLM	5.0	4.142046	0.44871795
BCAT1	5.0	14.470737	0.44871795
ILF3	5.0	99.44762	0.45454547
MID1IP1	4.0	3.8814538	0.42168674
PYGM	4.0	18.933332	0.43209878
RPL9	3.0	3.8888888	0.40697673
PHKB	2.0	3.2833333	0.3888889
AIMP2	2.0	5.815873	0.3723404
DTNBP1	1.0	0.0	0.34653464
LXN	1.0	0.0	0.3398058
CKAP4	1.0	0.0	0.3153153

Table S6. Genes ranked by degree method in sheep.

Gene	Degree	Betweenness	Closeness
PPARGC1A	15.0	195.56067	0.64
EPAS1	13.0	102.93597	0.6037736
IGF2	10.0	81.04654	0.5714286
MYOD1	10.0	70.97202	0.55172414
LOC443512	10.0	103.93407	0.53333336
THRB	9.0	34.33643	0.53333336
JUP	9.0	49.513065	0.53333336
APOA1	9.0	67.13152	0.5423729
JAG1	8.0	17.027718	0.5

CXCR4	8.0	30.54249	0.5
LOC780509	7.0	22.143147	0.5
ISG17	7.0	33.30916	0.4848485
TYMS	7.0	86.919495	0.47761193
DDIT3	6.0	12.685348	0.4923077
NAMPT	6.0	27.598701	0.4923077
ARG2	6.0	40.530468	0.4923077
BCAT1	6.0	36.136936	0.47058824
PLIN2	5.0	11.188095	0.47058824
UCP3	5.0	10.12381	0.4848485
POSTN	5.0	3.3403542	0.43835616
GCLM	5.0	5.441148	0.46376812
SDC4	5.0	8.9442005	0.47058824
ME1	5.0	13.105556	0.45070422
DGAT2	5.0	9.322222	0.43835616
PYGM	5.0	67.959526	0.46376812
EEF1A1	5.0	16.93956	0.43243244
HBB	4.0	3.8078144	0.46376812
MID1IP1	4.0	7.5968256	0.41558442
RPL9	4.0	4.7214284	0.41025642
ILF3	3.0	21.185715	0.43835616
FST	2.0	0.0	0.3902439
AIMP2	1.0	0.0	0.3265306
PHKB	1.0	0.0	0.32

Table S7. Genes ranked by degree method in pig.

Gene	Degree	Betweenness	Closeness
PPARGC-1	13.0	213.32454	0.6037736
ENSSSCP00000025976	13.0	203.44206	0.58181816
EPAS1	11.0	87.56209	0.58181816
JUP	10.0	88.565506	0.55172414
THRB	10.0	61.022007	0.5614035
NAMPT	8.0	37.424603	0.5
MYOD1	8.0	55.518803	0.52459013
CXCR4	8.0	49.640354	0.5
ME1	7.0	25.864286	0.46376812
JAG1	7.0	28.062302	0.4848485
BCAT1	7.0	16.571825	0.45714286
TYMS	7.0	82.67845	0.47058824
APOA1	7.0	71.82478	0.5
ISG15	6.0	39.80122	0.46376812

EEF1A1	6.0	58.69365	0.43243244
DDIT3	5.0	9.625	0.45714286
GCLM	5.0	4.510714	0.45070422
ARG2	5.0	16.216667	0.45070422
COL1A2	5.0	18.989317	0.42666668
SDC4	5.0	18.959036	0.43243244
PYGM	4.0	13.378205	0.44444445
POSTN	4.0	1.6617826	0.3764706
HBB	4.0	17.73602	0.45070422
ILF3	4.0	79.98468	0.43243244
UCP3	3.0	1.3333334	0.44444445
FST	3.0	2.0	0.3902439
PLIN2	3.0	2.9538462	0.42105263
PHKB	3.0	1.5	0.38554215
RPL9	3.0	2.4444444	0.37209302
S14-R	2.0	2.0916667	0.39506173
ENSSSCP00000026326	2.0	2.6188035	0.41025642
AIMP2	1.0	0.0	0.32323232
CKAP4	1.0	0.0	0.30476192
